# Complete genome sequence data of *Streptococcus pneumoniae* serotype 17F isolated in Malaysia

**DOI:** 10.1016/j.dib.2026.113264

**Published:** 2026-09-15

**Authors:** Revathy Arushothy, Cheng-Ngee Tan, Nazirah Samsudin, Rohaidah Hashim

**Affiliations:** a Institute for Medical Research, National Institutes of Health Malaysia, Ministry of Health Malaysia, Persiaran Setia Murni U13/52, Setia Alam, 40170, Shah Alam, Selangor, Malaysia; b Institute of Biological Sciences, Universiti Malaya, 50603, Kuala Lumpur, Malaysia

**Keywords:** *Streptococcus pneumoniae*, Serotype 17F, Whole genome sequencing, Genomic characterization, Malaysia

## Abstract

This study reports the genomic insights into a *Streptococcus pneumoniae* serotype 17F isolate in Malaysia (IMRCC/SS430/25), collected from an adult patient with aspiration pneumonia in 2025. Phenotypic identification revealed characteristic alpha-haemolytic colonies and optochin susceptibility. Species identity as S. pneumoniae was further verified by bile solubility testing and the 17F serotype was confirmed using the Quellung reaction. The isolate was subjected to whole-genome sequencing using the Oxford Nanopore platform. The assembly yielded a highly accurate, single circular contig of 2,065,967 bp with 100% completeness and 48× coverage. In silico typing assigned the genome to ST2355 within the globally disseminated GPSC10 cluster. Antimicrobial susceptibility testing demonstrated resistance to erythromycin, azithromycin, clindamycin, and tetracycline. This phenotypic profile was concordant with genomic screening which identified the *ermB* and *tetM_8* genes responsible for macrolide-lincosamide-streptogramin B (MLSB) and tetracycline resistance respectively. The penicillin-binding protein allelic profile (*pbp1a*: 79, *pbp2b*: 26, *pbp2x*: 194) predicted uniform susceptibility to beta-lactam antibiotics shown by phenotypic testing of amoxicillin-clavulanate and penicillin. Phylogenomic characterization identified the Malaysian isolate within the predominant global ST2355 clade of GPSC10, clustering alongside isolates from Nepal, the United Kingdom, Bangladesh, and the United States. This study demonstrates the utility of combining routine clinical microbiology with high-resolution genomic surveillance to resolve lineage relationship, reveal underlying resistance mechanisms, and confirm genotype-phenotype concordance.

Specifications Table

**Table:** 

Subject	Biology
Specific subject area	*Genomics.*
Type of data	Raw genome sequence data in FASTQ format.
Data collection	Genome sequencing platform: Oxford Nanopore Genomic assembly: EPI2ME wf-bacterialgenomes Genomic analysis: Pathogenwatch (https://pathogen.watch/) Parameters for data collection: Genomic DNA was extracted from purified cultures of *S. pneumoniae.* Description of data collection: Whole genome sequencing, assembly, annotation, genomic analysis
Data source location	Institute for Medical Research, National Institutes of Health Malaysia, Persiaran Setia Murni U13/52, Setia Alam, 40170 Shah Alam, Malaysia.
Data accessibility	Repository name: National Center of Biotechnology Information (NCBI) Data identification number: BioProject database: PRJNA1245582, Sequence Read Archive (SRA) database: SRR37999637 Direct URL to data: https://trace.ncbi.nlm.nih.gov/Traces?run=SRR37999637
Related research article	None

## 1 Value of the Data

• Data provides the genomic information of *S. pneumoniae* serotype 17F from a clinical isolate in Malaysia.
• Data characterise the antibiotic resistome of the *S. pneumoniae* serotype 17F strain and provide baseline information on its antimicrobial resistance burden in Malaysia.
• Data support broader genome studies of *S. pneumoniae* circulating among patients in Malaysia.

## 2 Background

*Streptococcus pneumoniae* (pneumococcus) is a major human pathogen that colonizes the upper respiratory tract, and causes both invasive and non-invasive diseases, contributing significantly to global morbidity and mortality [1]. *S. pneumoniae* was previously estimated to be the leading cause of non-COVID lower respiratory tract infections (LRTI) globally and in Malaysia [2]. To reduce disease burden, pneumococcal conjugate vaccines (PCVs) have been administered to children born since 2020 through the Malaysian National Immunization Programme (NIP) [3]. These vaccines target specific capsular serotypes, with PCV10 introduced in late 2020 and subsequently replaced by PCV13 in 2023. On the other hand, vaccination for adults involve both the 23-valent pneumococcal polysaccharide vaccine (PPV23) and 13-valent pneumococcal conjugate vaccine (PCV13) [3,4]. However, adult immunization is not mandatory, but only recommended based on age (adults aged 60 and older) or specific risk factors, including chronic or immunocompromising conditions and religious pilgrimages such as Hajj [3]. As part of the surveillance of circulating serotypes, this study reports the genomic characterization of a *S. pneumoniae* serotype 17F isolate from Malaysia, which is a serotype specifically covered by PPV23.

## 3 Data Description

A clinical isolate of *Streptococcus pneumoniae* serotype 17F (IMRCC/SS430/25) was identified in 2025 at the Bacteriology Unit, Institute for Medical Research (IMR), following recent observations indicating the emergence of serotype 17F in Malaysia. This isolate was subsequently selected for genomic characterization. The isolate was collected from a 46-year-old Malay male who suffered from aspiration pneumonia and was admitted to Hospital Sungai Buloh, Selangor, Malaysia. The pneumococcal isolate (IMRCC/SS430/25) was isolated from a tracheal aspirate specimen collected from the patient. The isolate displayed typical *S. pneumoniae* morphology when cultured on 5% Columbia Sheep Blood Agar, presenting as small, round, alpha-haemolytic colonies with an inhibition zone >14mm around an optochin disk (Fig. 1). Subsequent bile solubility testing confirmed the identity of *S. pneumoniae* while serotyping via the Quellung reaction using antisera from the Statens Serum Institut verified the isolate as serotype 17F.

**Fig. 1:**
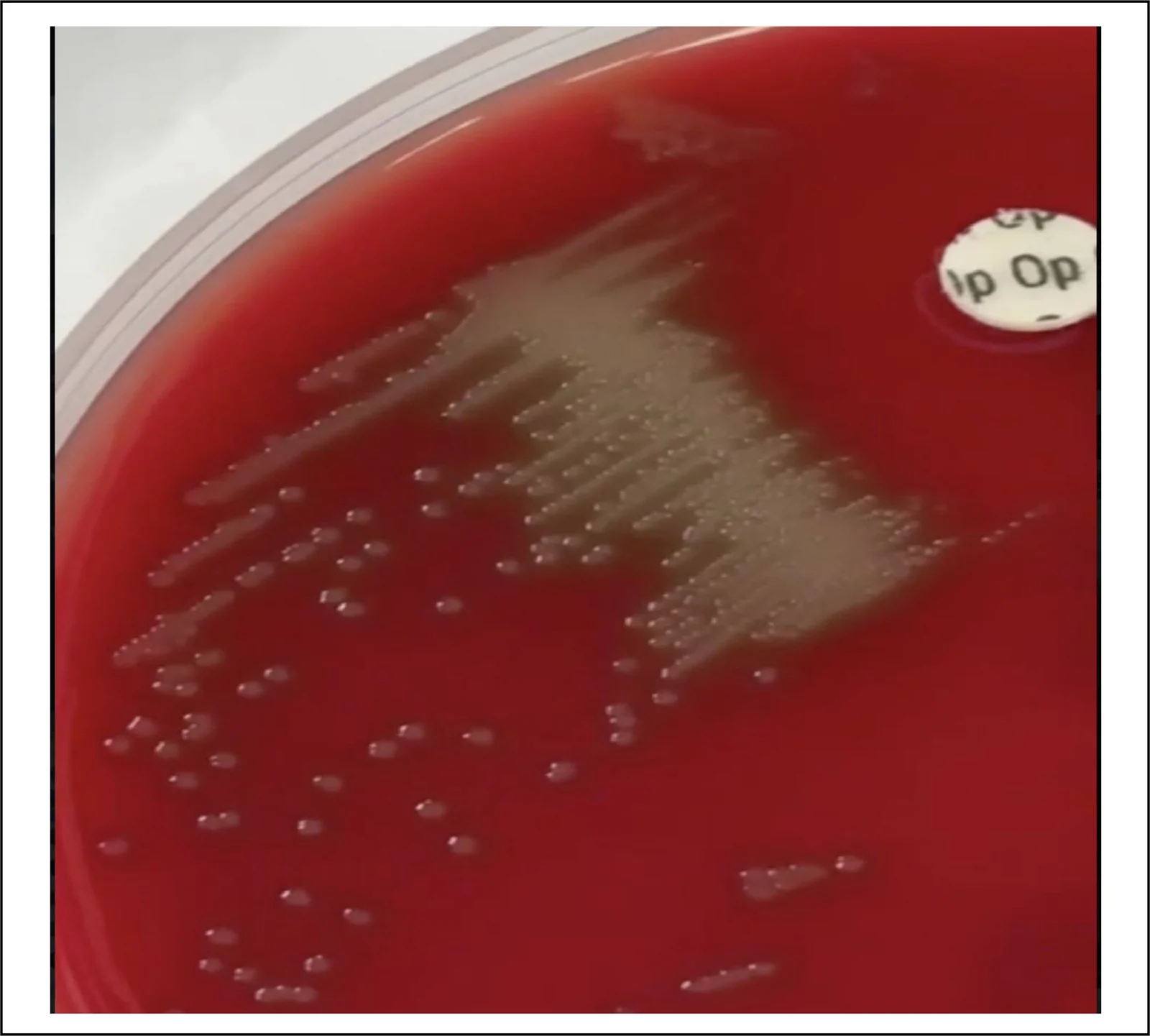
Streptococcus pneumoniae serotype 17F isolate colonies grown on 5% Columbia Sheep Blood Agar exhibited a greenish-brown, small, round appearance. A clear zone observed around optochin disc (>14mm) is a typical characteristic to identify S. pneumoniae.

Table 1 summarizes the Sequencing, assembly, annotation, and lineage information of the isolate. The pneumococcal isolate was subjected to genomic DNA extraction using the DNeasy® UltraClean® Microbial Kit (Qiagen, Germany) in accordance with the manufacturer’s protocol (HB-2248-003). Genomic DNA was subjected to quality control using the Qubit^TM^ dsDNA BR (Broad Range) Assay kit (Thermo Fisher Scientific, USA). Library preparation of the genomic DNA was performed using the Native Barcoding Kit 24 V14 (SQK-NBD114.24) following manufacturer’s protocol. The DNA library of the isolate was then subjected to whole genome sequencing (WGS) using the Oxford Nanopore MinION Mk1B platform. FASTQ data was subjected to quality control, which revealed a total of 108,286,605 bases were generated from 77,255 reads in the sequencing. The mean Phred score of the raw sequencing reads was 15.6, and the read length N50 for the sequenced data was 4,541 bp. The raw FASTQ reads were then used to assemble the pneumococcal genome through EPI2ME integrated workflow wf-bacterialgenomes. The assembly produced a single circular contig of pneumococcal genome (Fig. 2), with length of 2,065,967 bp and coverage of 48×. GC content of the genomes was 39.71%. Assessment of genomic quality through CheckM2 identified a 100% completeness and contamination of 0.23%. The genome annotation from Bakta revealed 2093 genes, including 2022 protein coding sequences (CDS), 12 rRNAs, 58 tRNAs, and 1 tmRNA. Genomic screening via Pathogenwatch confirmed the isolate IMRCC/SS430/25 was assigned to ST2355 and clustered within GPSC10.

**Table 1:** Sequencing, assembly, annotation, and lineage information of *S. pneumoniae* isolate IMRCC/SS430/25.

Isolate		IMRCC/SS430/25
Nucleotide Accession Number		SRR37999637
Raw FASTQ	Number of sequences	77,255
	Total data yield (bp)	108,286,605
	Average Phred score	15.6
	N50 (bp)	4,541
Genome assembly	Contigs	1
	Genome Length (bp)	2,065,967
	Sequencing coverage	48×
	GC content (%)	39.71
	Completeness (%)	100
	Contamination (%)	0.23
Genome annotation features	Protein coding sequences (CDS)	2022
	tRNA	58
	rRNA	12
	tmRNA	1
Lineage information	Sequence type (ST)	2355
	Global Pneumococcal Sequence Cluster (GPSC)	10

**Fig. 2:**
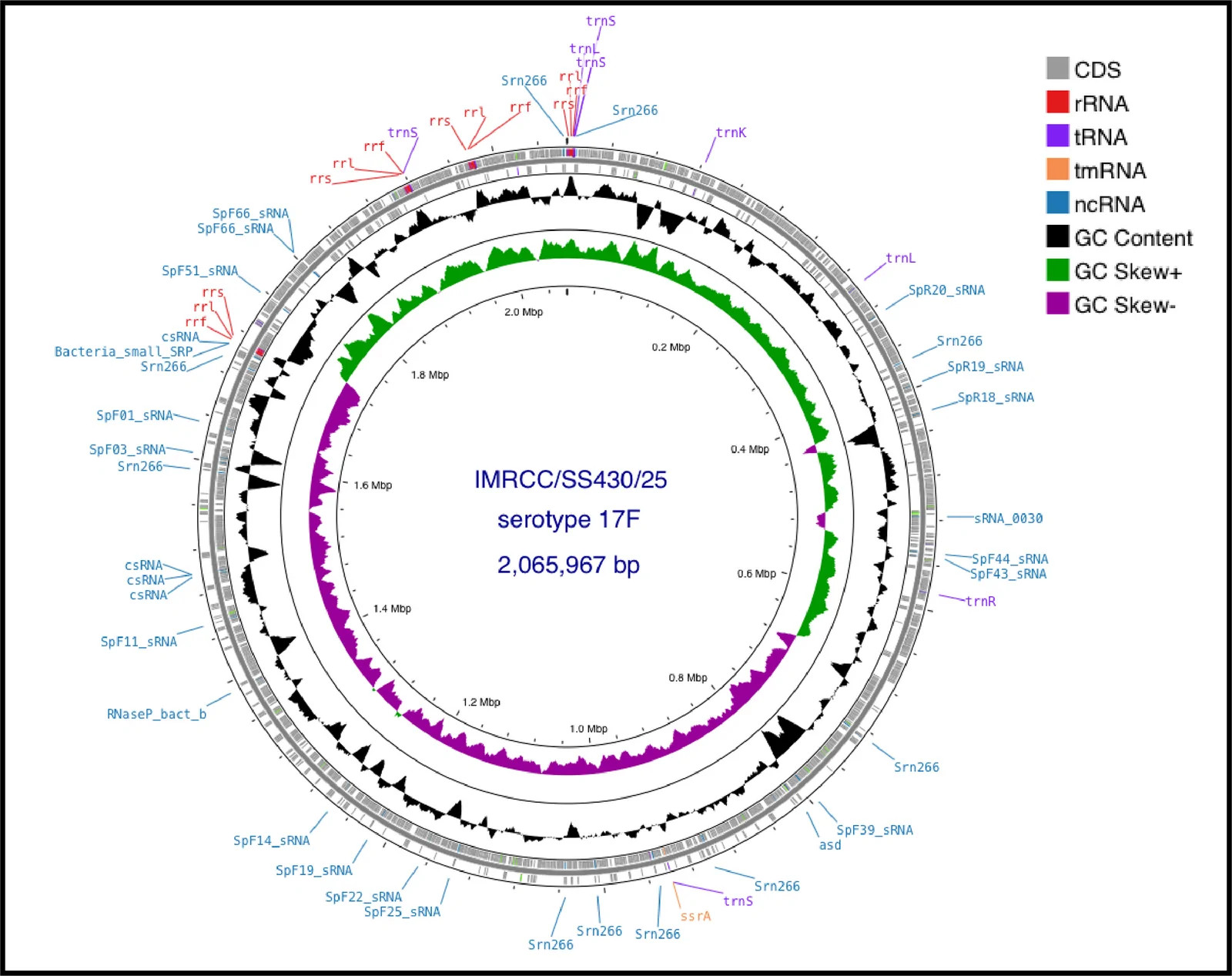
Circular genome map of S. pneumoniae serotype 17F isolate IMRCC/SS430/25. Tracks displayed from outermost to innermost: Bakta annotation on the forward and reverse strands for predicted coding sequences (CDS) (gray), ncRNA (blue), rRNA (red), tRNA (purple), tmRNA (orange), and predicted origin of replication (oriC, light green); GC content (black); and GC skew, with positive skew shown in green and negative skew in purple.

Antimicrobial susceptibility testing (AST) identified the serotype 17F isolate was resistant to erythromycin, azithromycin, clindamycin, and tetracycline (Table 2). Subsequent genomic analyses of resistance determinants through Pathogenwatch identified the *ermB* gene, which is responsible for macrolide-lincosamide-streptogramin B (MLS_B_) resistance, as well as the *tetM*_8 gene which confers tetracycline resistance. Interestingly, ST2355 genomes from Thailand and Israel were not detected with *ermB* gene in contrast to genomes of the same lineage from the USA and Canada [5]. Furthermore, IMRCC/SS430/25 was found to be susceptible to all beta-lactams tested in this study (penicillin, amoxicillin-clavulanate, cefotaxime) (Table 2). The penicillin-binding protein (*pbp*) allelic profile (*pbp1a*: 79, *pbp2b*: 26, *pbp2x*: 194) identified through Pathogenwatch analysis was predicted to confer uniform susceptibility to beta-lactams for IMRCC/SS430/25. This *in silico* prediction aligns with our phenotypic AST results, which demonstrated susceptibility to penicillin, cefotaxime, and amoxicillin-clavulanate [5].

**Table 2:** Antimicrobial susceptibility testing (AST), prediction of antimicrobial resistance determinants, and penicillin-binding protein (*pbp*) gene profiles of *S. pneumoniae* serotype 17F isolate IMRCC/SS430/25.

Isolate Genome	Antimicrobial susceptibility testing (AST)												Genotypic prediction			
	PEN		CTX		AMC	ERY	AZI	CLI	TET	CHL	SXT	VAN	Resistance Determinants	*pbp* gene profile		
	men	non-men	men	non-men										*pbp1a*	*pbp2b*	*pbp2x*
IMRCC/SS430/25	S	S	S	S	S	R	R	R	R	S	S	S	*ermB, tetM*_8	79	26	194

PEN G: Penicillin G, CTX: Cefotaxime, AMC: Amoxicillin-clavulanate, ERY: Erythromycin, AZI: Azithromycin, CLI: Clindamycin, TET: Tetracycline, CHL: chloramphenicol, SXT: trimethoprim-sulfamethoxazole, VAN: Vancomycin

men.: meningitis, non-men.: non-meningitis, S: sensitive, R: resistant

A maximum-likelihood phylogenomic tree rooted against reference strain TIGR4 positioned the Malaysian serotype 17F isolate (IMRCC/SS430/25) alongside 15 GPSC10 serotype 17F genomes (Fig. 3). The GPSC10 population was dominated by ST2355 (10/16 isolates), alongside five less frequent sequence types (ST17098, ST230, ST16399, ST11922, and ST8812). The Malaysian isolate, which was assigned to ST2355, clustered within the predominant ST2355 group together with isolates from several geographical regions. Other closely related sequence types, including ST11922, ST16399, and ST8812, were positioned within the broader ST2355-associated grouping, whereas ST17098 and ST230 formed a separate branch from the predominant ST2355 group. To further assess the allelic relatedness of these sequence types to the Malaysian isolate, the seven-locus MLST profiles were compared relative to ST2355 (Table 3). ST11922 and ST8812 were single-locus variants (SLVs) of ST2355, differing at one MLST locus each, while ST16399 was a double-locus variant (DLV), differing from ST2355 at two loci. In contrast, ST230 and ST17098 differed from ST2355 at three and five MLST loci, respectively, indicating greater allelic divergence from the Malaysian ST2355 isolate. These findings provide additional allelic context to the phylogenomic relationships observed within the GPSC10 serotype 17F population.

**Fig. 3:**
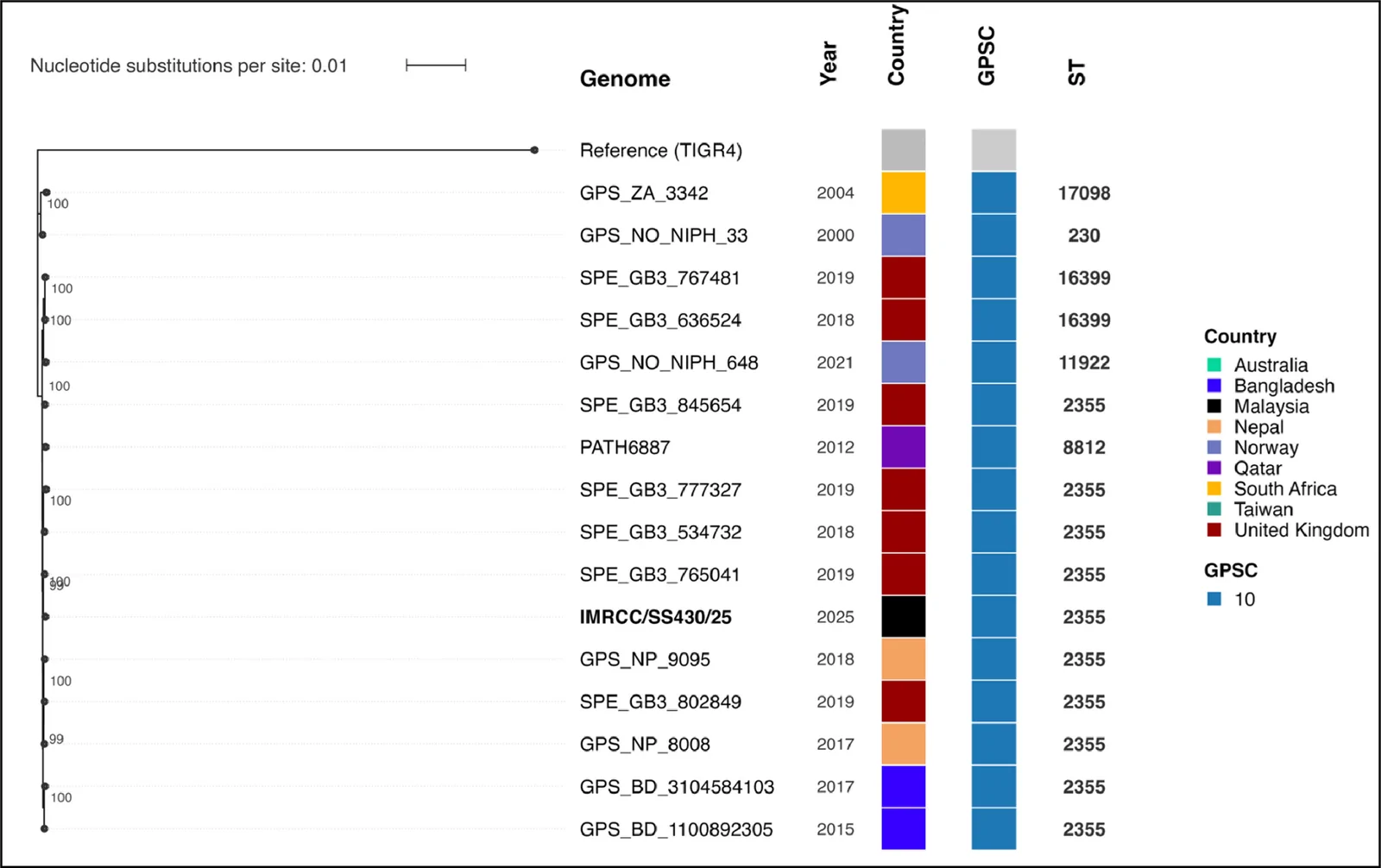
Maximum-likelihood phylogenetic tree comprising the Malaysian serotype 17F isolate IMRCC/SS430/25 in comparison to 15 global serotype 17F S. pneumoniae genomes. The tree was rooted using the TIGR4 reference genome. Numbers at internal nodes represents ultrafast bootstrap (UFBoot) support value. Tip labels are annotated from left to right with the year of isolation, country, Global Pneumococcal Sequence Cluster (GPSC), and sequence type (ST). Country and GPSC are indicated by color, while STs are indicated numerically. The Malaysian isolate IMRCC/SS430/25 from this study is highlighted in bold.

**Table 3:** Seven-locus MLST allelic profiles and relationships among GPSC10 serotype 17F sequence types (STs) and their relationship to ST2355.

ST	*aroE*	*gdh*	*gki*	*recP*	*spi*	*xpt*	*ddl*	Relationship to ST2355
**2355**	2	19	2	17	15	22	17	Malaysian isolate
11922	2	19	2	17	15	22	589	SLV
8812	2	19	2	17	15	22	8	SLV
16399	2	19	2	17	684	22	589	DLV
230	12	19	2	17	6	22	14	3-locus variant
17098	12	745	2	1	6	22	14	5-locus variant

ST, sequence type; MLST, multilocus sequence typing; SLV, single-locus variant; DLV, double-locus variant. Allelic relationships are shown relative to ST2355 (IMRCC/SS430/25). SLVs and DLVs differ from ST2355 at one and two MLST loci, respectively.

## 4 Experimental Design, Materials and Methods

### 4.1 Bacterial culture, identification, and serotyping

The *S. pneumoniae* serotype 17F isolate (IMRCC/SS430/25) was previously archived by the Institute for Medical Research (IMR) Malaysia. The archived isolate was first cultured on 5% Columbia Sheep Blood Agar and incubated in 5% CO2 at 37°C for 18 to 24 hours. The isolate was evaluated for colony morphology, alpha-hemolysis pattern, and optochin susceptibility. A bile solubility test using 10% sodium deoxycholate was performed to observe the clearing of turbid suspension which indicated the identity of *S. pneumoniae*. The serotype of isolate was then confirmed via Quellung reaction using serotype/serogroup specific antisera (SSI Diagnostica, Denmark).

### 4.2 Antimicrobial susceptibility testing

The pneumococcal isolate was subjected to antimicrobial susceptibility using the Epsilometer test for penicillin G, cefotaxime, and amoxicillin-clavulanate, while disk diffusion method was used for, erythromycin, azithromycin, clindamycin, tetracycline, chloramphenicol, trimethoprim-sulfamethoxazole, and vancomycin. Susceptibility categories for each antimicrobial agents were interpreted in accordance with the 36^th^ edition Clinical and Laboratory Standards Institute (CLSI) Performance Standards for Antimicrobial Susceptibility Testing guidelines [6].

### 4.3 DNA extraction and library preparation

Genomic DNA of the *S. pneumoniae* isolate was extracted using the DNeasy® UltraClean® Microbial Kit (Qiagen, Germany) following the manufacturer’s protocol (HB-2248-003). DNA concentration was measured using the Qubit dsDNA Broad Range assay (Thermo Fisher Scientific, USA) according to the manufacturer’s protocol (Pub. No. MAN0002325 Rev. B.0). Library preparation of the genomic DNA was performed using the Oxford Nanopore Native Barcoding Kit 24 V14 (SQK-NBD114.24) using 400 ng of input DNA, according to the manufacturer’s protocol (NBA_9168_v114_revT_02Oct2025). Long read sequencing was performed on the MinION Mk1B sequencing platform using the MinKNOW software (version 25.09.16), with at least 40× sequencing coverage of the genome size.

### 4.4 Sequencing analysis

Raw sequence data was basecalled in real-time using Dorado (version 1.3.0) and its integrated super-accurate basecalling (SUP) model (v5.2.0) (Oxford Nanopore Technologies Ltd., UK) [7]. The resulting raw FASTQ data was filtered during the basecalling to exclude reads with mean Phred quality score below Q10 to ensure an accurate, high-fidelity FASTQ output for downstream analysis. Quality of the FASTQ reads was then assessed using NanoPlot (version 1.46.2) [8]. *De novo* genome assembly was performed using the EPI2ME integrated workflow wf-bacterialgenomes (https://github.com/epi2me-labs/wf-bacterial-genomes). The pipeline automates the assembly of reads using Flye (version 2.9.6), contig reorientation via dnaapler (version 1.3.0), and alignment with mini_align (Pomoxis version 0.3.6, (https://nanoporetech.github.io/pomoxis/programs.html#mini-align) which is based on minimap2 (version 2.30). Subsequent polishing and coverage estimation were performed using Medaka (version 2.2.0) (https://github.com/nanoporetech/medaka), and mosdepth (version 0.3.12) (https://github.com/brentp/mosdepth) respectively [[9], [10], [11], [12]]. The quality of the assembled genome was examined through CheckM2 (version 1.1.0) to assess genome completeness and evaluate potential contamination [13]. Genome annotation was performed on Bakta (version 1.12.0) and was visualised using Proksee as a circular map [14,15]. The assembled *S. pneumoniae* genomes were then subjected to further analysis using Pathogenwatch (version 23.5.2) (https://pathogen.watch). This platform was utilized to determine Sequence Type (ST), Global Pneumococcal Sequence Cluster (GPSC), and the presence of antimicrobial resistance (AMR) genes, including specific variations in resistance genes and penicillin-binding protein (*pbp*) alleles [5]. A phylogenetic analysis was performed to compare the Malaysian serotype 17F isolate within the global GPSC10 serotype 17F population from Monocle (https://data-viewer.monocle.sanger.ac.uk/project/gps). The phylogenomic tree was constructed using Snippy (version 3.2.0) and IQ-TREE (version 3.1.1) and subsequently visualized using R package ggtree [[16], [17], [18]]. Based on the STs of the genomes included in the phylogenomic analysis, the allelic profiles of the seven MLST loci for each ST were retrieved from PubMLST (https://pubmlst.org), and the number of differing loci between STs was determined to assess pairwise genetic relatedness [19].

## Limitations

The study provides a limited representation of *Streptococcus pneumoniae* serotype 17F in comparison to the Malaysian population. Besides, the estimation of sequencing depth may only represent the average genome coverage, which does not exclude the possibility of lower coverage across certain genomic regions.

## Ethics Statement

This study was approved by the Malaysian Research Ethics Committee and National Medical Research Registry under the protocol number NMRR-24-01370-HHE, following The Code of Ethics of the World Medical Association (Declaration of Helsinki).

## CRediT Author Statement

**Revathy Arushothy:** Supervision, Conceptualization, Methodology, Writing-Original Draft, Writing-Review and Editing, **Cheng-Ngee Tan:** Investigation, Formal analysis, Visualization, Writing-Original Draft, **Nazirah Samsudin:** Investigation, Resources, **Rohaidah Hashim:** Project administration, Supervision, Resources.
